# Surface Modification of Cellulose Nanocrystals with Succinic Anhydride

**DOI:** 10.3390/polym11050866

**Published:** 2019-05-13

**Authors:** Agnieszka Leszczyńska, Paulina Radzik, Ewa Szefer, Matej Mičušík, Mária Omastová, Krzysztof Pielichowski

**Affiliations:** 1 Department of Chemistry and Technology of Polymers, Cracow University of Technology, ul. Warszawska 24, 31-155 Kraków, Poland; paulinaradzik@indy.chemia.pk.edu.pl (P.R.); eszefer@chemia.pk.edu.pl (E.S.); kpielich@pk.edu.pl (K.P.); 2 Polymer Institute, Slovak Academy of Sciences, Dúbravská cesta 9, 845 41 Bratislava 45, Slovakia; matej.micusik@savba.sk (M.M.); Maria.Omastova@savba.sk (M.O.)

**Keywords:** cellulose nanocrystals, whiskers, surface modification, esterification, succinic anhydride

## Abstract

The surface modification of cellulose nanocrystals (CNC) is a key intermediate step in the development of new functionalities and the tailoring of nanomaterial properties for specific applications. In the area of polymeric nanocomposites, apart from good interfacial adhesion, the high thermal stability of cellulose nanomaterial is vitally required for the stable processing and improvement of material properties. In this respect, the heterogeneous esterification of CNC with succinic anhydride was investigated in this work in order to obtain CNC with optimised surface and thermal properties. The influence of reaction parameters, such as time, temperature, and molar ratio of reagents, on the structure, morphology and thermal properties, were systematically studied over a wide range of values by DLS, FTIR, XPS, WAXD, SEM and TGA methods. It was found that the degree of surface substitution of CNC increased with the molar ratio of succinic anhydride to cellulose hydroxyl groups (SA:OH), as well as the reaction time, whilst the temperature of reaction showed a moderate effect on the degree of esterification in the range of 70–110 °C. The studies on the thermal stability of modified nanoparticles indicated that there is a critical extent of surface esterification below which only a slight decrease of the initial temperature of degradation was observed in pyrolytic and oxidative atmospheres. A significant reduction of CNC thermal stability was observed only for the longest reaction time (240 min) and the highest molar ratio of SA:OH. This illustrates the possibility of manufacturing thermally stable, succinylated, CNC by controlling the reaction conditions and the degree of esterification.

## 1. Introduction

Cellulose nanocrystals, or CNCs, have recently emerged as a new class of versatile building blocks delivered from renewable feedstock [1]. Due to their high mechanical strength, low density, functionalisability, environmental sustainability and low cost, they have been extensively explored as a reinforcement in polymer nanocomposites [2,3]. However, the limited thermal stability of cellulose nanoparticles is a significant problem in nanocomposite manufacturing. Thus, extensive work has already been carried out to obtain thermally stable cellulose nanocrystals [4].

The most commonly used method of CNC preparation is the hydrolysis of cellulose materials in mineral acids. According to the literature, sulphuric acid has the widest application in cellulose hydrolysis [5,6,7,8,9]. For example, Beck-Candanedo et al. investigated the influence of reaction time and sulphuric acid-to-pulp ratio on nanocrystal properties, and showed that longer hydrolysis times produced shorter, less polydisperse cellulose nanocrystals, and furthermore, increasing the acid-to-pulp ratio caused a reduction of CNC dimensions [10]. Since cellulose nanocrystals obtained from sulphuric acid are characterised by a relatively low thermal stability, other mineral and organic acids have been used to produce CNCs. Amongst mineral acids, the application of phosphoric acid allowed for the production of nanocrystalline cellulose with higher thermal stability in comparison to sulphuric acid hydrolysis [4,11]. Other approaches employed in cellulose hydrolysis included, for example, application of hydrochloric acid [12,13], a mixture of acetic and nitric acid [14], and formic or hydrobromic acid [15,16,17].

One of the main applications predicted for CNCs is the manufacturing of polymeric nanocomposites. CNCs often require previous surface modification in order to achieve good compatibility with a polymer matrix and a better individualisation of nanoparticles. Alongside methods such as silylation or acylation, the carboxylation of cellulose nanocrystals leads to their better compatibility with hydrophobic thermoplastic matrices, and moreover it was shown that the yield of nanoparticles increases with the carboxyl group content for a series of TEMPO-oxidized samples [18,19]. The introduction of charged groups and the reduction of CNC hydrophilicity, by decorating their surface with less-polar organic moieties, has been widely reported. For example, Hu et al. obtained carboxylated cellulose nanocrystals with negatively charged carboxylic groups from borer powder of bamboo by direct oxidative degradation with ammonium persulfate [20]. Surface carboxylated cellulose nanocrystals, with repelling forces between individual nanocrystals, were also prepared via 2,2,6,6-tetramethylpiperidine-1-oxyl(TEMPO)-mediated oxidation of cotton linters by Montanari et al. [21]. Another approach for preparing carboxycellulose was described by Sharma et al. who used nitric acid or nitric acid-sodium nitrite mixtures for this purpose, and this method was shown to be less energy and water intensive compared to the TEMPO oxidation of cellulose [22]. The most common chemical methods of CNC surface functionalisation with organic substituents are esterification, etherification and grafting [1]. Chemical derivatisation of cellulose nanomaterial is likely to diminish its thermal stability, which is another critical factor for the processing of polymeric nanocomposites. However, the improved thermal stability of nanocellulose was reported, for example, by Yin et al. as a result of triazine derivative grafting on the cellulose surface to replace hydroxyl groups [23,24]. 

Furthermore, the decorating of nanocellulose with functional organic moieties provides nanoparticles with the desired reactivity in further processing steps. Amine functionalised CNCs were obtained by Yue et al. using amino trimethoxy silane. Functionalisation of the filler allowed a further reaction with a bio-based epoxy resin, derived from diphenolic acid, to manufacture bio-based epoxy hybrid nanocomposites [25]. 

Besides using CNCs as a reinforcement in polymer nanocomposites, other applications of modified cellulose nanocrystals are demonstrated in the literature. Hydrophobisation of cellulose nanocrystals allows them to be used as coating agents for marine vehicles, biomedical devices, windows, textiles or paints [26]. Another possibility is to use CNCs as antimicrobial agents, which is of great interest to the food packaging industry. For example, antimicrobial activity against *E. coli* was reported by Tang et al. and Drogat et al. for cellulose nanoparticles with polyrhodanine, or composites of silver nanoparticles and CNCs, respectively [19,26,27,28]. Also Sharma and Varma showed that agricultural residue derived cellulose and cotton cellulose can be used for producing quasi-spherical shaped nanoparticles with improved anti-microbial properties for *E. coli* [29]. Cellulose nanocrystals have also been recently investigated for their use in water treatment technologies, e.g., phosphorylated CNC has promoted heavy metal adsorption, such as Ag^+^, Cu^2+^ and Fe^3+^ from industrial effluents [30,31,32]. Other applications of nanocrystalline cellulose include support for catalysts, emulsion stabilisers and flocculating agents [19].

Succinic anhydride can be considered a promising modifying agent for nanocellulose since it enables the introduction of aliphatic chains along with carboxylic end groups. Thus, its chemical structure is useful in the engineering of physical and chemical surface properties of cellulose nanomaterial. Carboxylic functional groups provide surface charges and reactivity in further derivatization of the nanoparticles. Moreover, significant progress has been reported for the production of succinic acid from renewable resources as a platform intermediate of high significance [33]. In the bio-economy era, the application of renewable chemical feedstock with good availability is critical for the successful application of bionanomaterials. In this respect, succinic anhydride is advantageous in specific applications over, e.g., acid chlorides or common silylation agents, despite the high reactivity of the latter.

The application of succinic anhydride in the chemical derivatisation of carbohydrates has been reported for starch, chitosan and cellulose [34]. However, there are few reports on the functionalisation of cellulose nanoparticles with succinic acid or anhydride and its derivatives, and they are rather focused on studying the functional properties of modified nanoparticles and composite materials. Esterification of raw cellulose with succinic anhydride has been demonstrated as a very effective method of surface charging that facilitated defibrillation in a microfluidizer [35]. Succinic acid was applied by Castro et al. as a bridging agent to attach β-cyclodextrin to the surface of cellulose nanocrystals. As a result, new materials were produced with the ability to release antibacterial molecules over a prolonged period of time [36]. Cellulose nanocrystals, decorated with succinate groups, also work as an effective nanoadsorbent for Pb^2+^ and Cd^2+^ heavy metal cations [37]. Moreover, carboxycellulose nanoparticles or zinc oxide nanocrystal-decorated microfibrillated cellulose scaffolds were used for water treatment applications, to remove, e.g., lead, arsenic or cadmium(II) [38,39,40,41]. Elsewhere, cellulose nanocrystals have been modified with alkenyl succinic anhydride (ASA) and changes in surface energies, and its influence on CNC dispersibility in polypropylene and polylactide matrices were studied [42]. Miao et al. discovered a high increase of storage modulus (487-fold increase) when ASA-modified cellulose nanocrystals were applied as a reinforcement to the polyurethane system [43]. 

Surface modification of cellulose nanocrystals has been intensively investigated to reach better compatibility of CNC with polymer matrix, and, in consequence, to achieve better properties of the composite materials. Although there are reports on CNC modification by succinic anhydride, we aimed at an elaboration of the optimal reaction conditions to achieve thermally stable succinylated CNC that is vital for its further application as a modifier for polymers processed at higher temperatures, by e.g., extrusion.

In the previous work we found that at lower concentrations of phosphoric acid (in the range of 60–74 wt.%) and at a lower temperature (90 °C), the main product of hydrolysis of UFC 100 cellulose was fine microcrystalline cellulose that was further used as a substrate for the production of CNCs in the presence of succinic anhydride without the use of esterification catalyst [4]. In such conditions, further hydrolysis of amorphous regions of cellulose as a dominating process and slight surface esterification were simultaneously running. In the current work we have optimised the phosphoric acid concentration and hydrolysis temperature at higher values for the production of thermally stable cellulose nanocrystals from different raw material, and then produced CNCs were subjected to surface modification with succinic anhydride (SA) in the presence of pyridine catalyst. We report the systematic study on the effects of various reaction parameters in the heterogeneous esterification of CNCs, in particular, the influence of reaction time, temperature and molar ratio of SA to hydroxyl groups of cellulose, on the structure, morphology and thermal properties of modified cellulose nanocrystals. The influence of phosphoric acid concentration on the properties of CNCs was discussed in reference to crystallinity changes as well as its thermal and thermo-oxidative stability.

## 2. Materials and Methods

### 2.1. Materials

Microcrystalline cellulose powder Avicel PH-101 from Sigma Aldrich (Darmstadt, Germany) was used as a starting material for further hydrolysis and modification. The average particle size of this material is 50 μm. Reagent grades of phosphoric acid solution (85 wt.%), dimethylformamide, pyridine and succinic anhydride were also purchased from Sigma Aldrich and used in the as received condition. Analytical grade sodium hydroxide supplied by Chempur (Piekary Śląskie, Poland) was used to prepare the solution for alkali treatment of the starting material. 

### 2.2. Preparation of Cellulose Nanocrystals

A total of 10 g of microcrystalline cellulose was added to a round-bottomed flask containing 200 mL of sodium hydroxide solution with a concentration of 4 wt.% and the suspension was stirred for 2 h at 80 °C. This process was repeated twice, with subsequent washing of the residue with distilled water to pH 7. Alkali treatment of cellulose was performed to remove non-cellulosic components present in the starting material [44]. The hydrolysis conditions were selected on the basis of the work by Espinosa et al. [45]. After alkali treatment, 1 g of dried microcrystalline cellulose was added to a round-bottomed flask and mixed with distilled water. The mixture was cooled in an ice bath and phosphoric acid (85 wt.%) was slowly added via a dropping funnel, keeping the temperature below 30 °C. The acid concentrations used in hydrolysis were 70, 74, 76 and 78 wt.%. The hydrolysis was carried out at 100 °C for 90 min under magnetic stirring. Subsequently, suspensions were diluted one-fold with distilled water to stop the hydrolysis and cooled down to room temperature in an ice bath. The residue of the prepared cellulose nanocrystals (CNC_P series) was separated from the acid solution by thrice repeated centrifugation (at 5200 rpm for 15 min), the supernatant being replaced with an equal volume of distilled water before undergoing ultrasonic homogenisation (using Sonic Ruptor 250 (Omni International, Kennesaw, GA, USA) ultrasonic homogeniser working at 50% of maximum amplitude, in a pulse mode) after each centrifugation step. After the third cycle of centrifugation and sonication, the suspension was placed in a dialysis membrane (MWCO 14 000) and dialysed against distilled water for five days. When the pH value of the suspension reached 7, it was sonicated for 15 min and five drops of ethanol were added to reduce the crystallisation of water and agglomeration of CNCs during drying. Then the dispersion was frozen with liquid nitrogen and lyophilised using FreeZone Plus 2.5 freeze-dryer (Labconoco, Kansas City, MO, USA).

**Figure 1:**
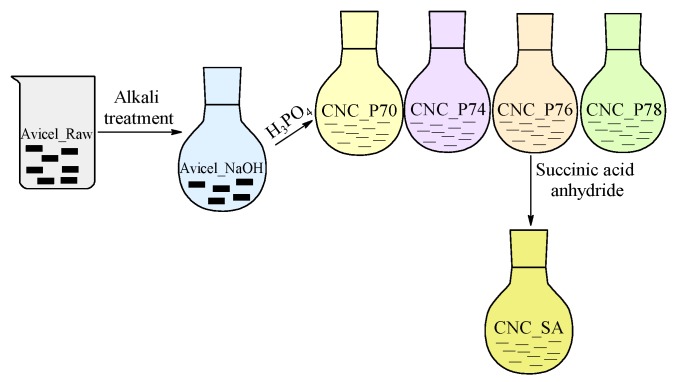
Schematic illustration of the preparation route of modified cellulose nanocrystals.

Cellulose nanocrystals obtained from hydrolysis in phosphoric acid at a concentration of 76 wt.% (CNC_P76) were then modified with succinic anhydride (SA) under a range of reaction conditions. The influence of the reaction time (60, 120, 180 and 240 min), temperature (70, 80, 90, 100 and 110 °C) as well as molar ratio of succinic anhydride to total cellulose hydroxyl groups (SA:OH 2:1, 3:1, 5:1 and 10:1) on the properties of modified CNCs was investigated. In each reaction, 0.5 g of CNC_P76 was dispersed in 16 mL of dimethylformamide (DMF) and after the reaction temperature was achieved, the SA solution in 10 mL of DMF and 0.026 mL of pyridine catalyst were added into the CNC dispersion. The catalyst type and amount was determined according to the work by Sehaqui et al. [35]. Reactions were carried out under nitrogen flow. After the reaction, the mixture was cooled down in an ice bath, centrifuged twice (5200 rpm, 15 min) and sonicated (15 min) with distilled water. To purify the CNC suspension from the remaining reagents, it was dialysed against distilled water for three days. In order to reduce the crystallisation of water and aggregation of CNC during freeze-drying, tert-butanol was added to the water dispersion of CNCs to achieve *t*-but:H_2_O 5:25 (*v*/*v*) concentration [46]. Finally, the CNCs dispersion was frozen in liquid nitrogen and freeze-dried. The preparation route of modified CNCs is presented in Figure 1 and denotations of all samples are shown in Table 1. If not indicated in the sample name as a varying parameter, the following standard parameters were applied: 110 °C, 120 min and 5:1, respectively for the reaction temperature, time and molar ratio of succinic anhydride to hydroxyl groups of cellulose (SA:OH).

### 2.3. Dynamic Laser Scattering (DLS)

Dynamic laser scattering (DLS) measurements were undertaken to determine particle-size distributions by intensities and numbers. A Malvern Zeta Sizer Nano ZS was used (Malvern, UK), and analyses were carried out in disposable sizing cuvettes, at a scattering angle of 173° and room temperature. Concentration of each dispersion was 0.1 mg·mL^−1^. The hydrodynamic radius, r_h_, derived from Stokes-Einstein equation for spherical particles, was determined by DLS method and was considered only as an indicative measure of the ‘apparent’ size of the dynamic hydrated/solvated CNCs. The intensity size distributions were obtained from analysis of the correlation function using the CONTIN algorithm in the instrument software.

### 2.4. Fourier Transform Infrared (FTIR)

Fourier transform infrared (FTIR) spectra were collected in the Spectrum 65 spectrophotometer (Perkin-Elmer, Waltham, MA, USA) in ATR mode with a C/ZnSe crystal. The range of each measurement was 4000–450 cm^−1^ in 4 cm^−1^ intervals. Eight scans were averaged.

### 2.5. X-ray Photoelectron Spectroscopy (XPS)

X-ray photoelectron spectroscopy (XPS) analysis was performed using a K-Alpha XPS system (Thermo Scientific, Waltham, MA, USA) equipped with a micro-focused, monochromatic Al Kα X-ray source (1486.6 eV). An X-ray beam of 400 μm size was used at 6 mA × 12 kV. The spectra were acquired in the mode of constant analyser energy and the pass energy was 200 eV for the survey. The fractional concentration of a respective element A (in atomic%) was calculated according to Equation (1):

(1) $$\%A=\frac{I_{A}/s_{A}}{\sum^{\;}(I_{n}/s_{n})}\cdot 100\%$$

where *I*_n_ is the integrated peak area of the detected atoms and *S*_n_ is the Scofield sensitivity factor corrected for the analyser transmission. The determined surface compositions were used to calculate the surface substitution degree (SSD) of cellulose. The method proposed by Rodionova et al. was adapted to perform the calculations [47]. It was considered that for each cellulose unit there are five carbons in the (C–O) molecular environment. Therefore, ⅕ of the (C–O) percentage peak contribution (C_%C–O_) corresponds to the contribution of a single carbon atom in the (C–O) environment. Since the cellulose was esterified with dicarboxylic acid anhydride, ½ of the percentage concentration of carbons in the (O–C=O) environment (C_%O–C=O_) was proportional to the number of ester bonds. The surface substitution degree (SSD) was calculated using Equation (2), as a concentration of ester bonds related to the concentration of a single (C–O) group:

(2) $$SSD=\frac{1}{2}C_{\%O–C=O}/\frac{1}{5}C_{\%C–O}$$

### 2.6. Wide-Angle X-ray Diffraction (WAXD)

X-ray diffraction measurements were performed using a D2 Phaser (Bruker, Billerica, MA, USA) diffractometer at room temperature in the reflection mode (k_Cu_ = 1.54 Å). Patterns were collected in the range of 5°–40° using an increment of 0.02°. The degree of crystallinity – χ_c_, was determined as the ratio of the total area of the crystalline peaks (Σ_Acrystal_) to the sum of the areas of the crystalline peaks and the amorphous profile (Σ_Aamorphous_). Deconvolution procedure was done using a MagicPlot Student 2.7.1. Five crystalline peaks were found at 14.3°, 16.4°, 20.0°, 22.5° and 34.5° which correspond to the 101, 10ī, 021, 002 and 040 Miller indices of cellulose crystals respectively [48,49,50].

### 2.7. Scanning Electron Microscopy (SEM)

Morphology of the samples was examined using a JSM-6010LA Jeol Scanning Electron Microscope (Jeol, Tokyo, Japan). The samples were metalized with gold-palladium using a Sputtering System Hummer 6.2 (Anatech USA, Hayward, CA, USA) with the thickness of the layer being approximately 3 nm.

### 2.8. Atomic Force Microscopy (AFM)

The AFM imaging of CNCs dried on air was carried out in tapping mode on AFM INNOVA (Veeco, New York, NY, USA). The Bruker’s antimony (n) doped Si AFM probe model FESPA-V2 with k value of 22.8 N/m and resonance frequency (*f*_0_) of 75 kHz was used at scanning rate of 0.300 µm/s. Typical topography and phase images were acquired. The CNC diameters were evaluated in the Bruker NanoScope Analysis 1.40 software by the determination of horizontal distances between valleys on the cross-sectional profiles taken perpendicular to the cellulose crystals. Thirty values of CNC diameters were averaged for each analysed sample.

### 2.9. Thermogravimetric Analyses (TGA)

Thermogravimetric analyses (TGA) were carried out using a TG F1 Libra analyser (Netzsch, Selb, Germany), and were collected in a temperature range of 30–600 °C, at a heating rate of 10 °C·min^−1^, under a synthetic air and argon atmosphere. 

## 3. Results and Discussion

### 3.1. Particle Size Distribution by DLS Measurements

**Figure 2:**
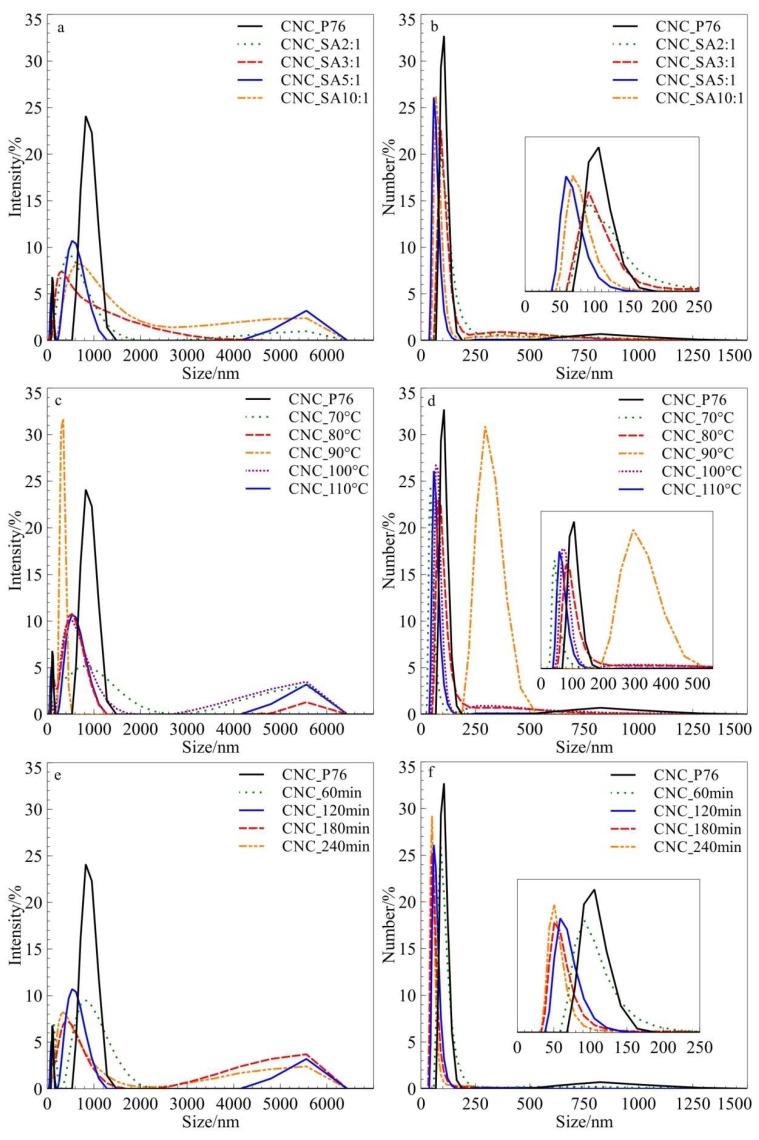
Particle size distribution for CNC_SA obtained at (**a**,**b**) different SA:OH molar ratios; (**c**,**d**) temperatures; and (**e**,**f**) time of reaction by intensity and number, respectively.

The dynamic light scattering (DLS) method was applied as a supporting technique for the evaluation of particle size distribution of CNC_P76 and succinylated nanocrystals. The investigated materials are polydisperse nanoparticles with a high aspect ratio. Therefore, the determined hydrodynamic radius was considered as a tentative measure of an ‘apparent’ size of the dynamic hydrated/solvated rod-like particles. Recently, Fraschini et al. demonstrated that a slight change in the length of CNC directly affected the measured DLS size, while changes in the cross-section showed hardly no effect on the diffusion velocity [51]. Also, according to Du et al., hydrodynamic diameters based on DLS measurements represents the length rather than the width of CNC samples [15]. Moreover, the DLS measurements show significantly higher sensitivity to bigger particles when intensity is plotted against size since a small number of bigger particles may cause significant scattering. Therefore, analysis of particle number versus size is more informative of the actual trends in size distribution in the modified samples. It has to be stressed that the fundamental size distribution generated by DLS is an intensity distribution, which can be converted, using Mie theory, to a volume distribution, and further to a number distribution. However, number distributions are of minor use since small errors in gathering data for the correlation function will lead to significant errors in distribution by number. Thus, due to limitations in the application of the DLS techniques for both optically and geometrically anisotropic nanoparticles presenting non-homogeneous size distribution, and the influence of larger particles, DLS measurements of cellulose nanocrystals were applied for assessing the relative size trends among the prepared series, not the exact sizes and size distribution. On the other hand, due to fast measurements providing qualitative information, DLS was considered as a convenient method providing useful technological information. DLS analysis of untreated and modified CNCs water suspensions showed that both the CNC_P76 and modified cellulose nanocrystals showed a bimodal distribution of particle sizes by intensity. CNC_P76 was composed of small nanoparticles with apparent hydrodynamic diameter in the range of 100–150 nm and the second fraction in the submicronic range of c.a. 0.6–1.4 µm. Modified CNCs showed a small fraction at around 5–6 μm in addition to nanometric fraction. With the increasing molar ratio of succinic anhydride to the hydroxyl groups of cellulose, the sizes of cellulose nanocrystals decreased. The same trend was observed for increasing the time of reaction. However, no clear dependence of the reaction temperature on the particles sizes was found, which can be seen in Figure 2. All modified samples, except the one modified at 90 °C, had smaller particle sizes of the nanometric fraction, with the hydrodynamic diameters in the range of 50–100 nm, compared to the sample after hydrolysis in the phosphoric acid, in which most of the particles had hydrodynamic diameters in the range of 100–150 nm. Such a result indicates that during the modification reaction of cellulose nanocrystals with succinic anhydride, further hydrolysis proceeds parallel to the esterification reaction. The microparticles were hardly visible when size distribution by a number of particles was considered. This can indicate a presence of a small amount of agglomerates or microparticles in the samples. The possible origin of the agglomerated particles is the freeze-drying of the produced CNC_P76 sample before succinylation. The proper drying was necessary for the exact control of cellulose mass, and thus, molar ratios during the esterification. A chemical crosslinking of particles after modification with the bifunctional succinic acid molecule is another considered effect that could lead to formation of bigger particles. The average length of nanoparticles gradually diminished with the increase of SA:OH molar ratio. When analysing the changes in size distribution versus reaction time, one can observe that CNC kept its original length after the first 60 min of esterification and underwent the most significant variation in sizes after 120 min. A further increase in esterification time had little effect on the CNC dimensions.

### 3.2. Structure Analysis by FTIR

**Figure 3:**
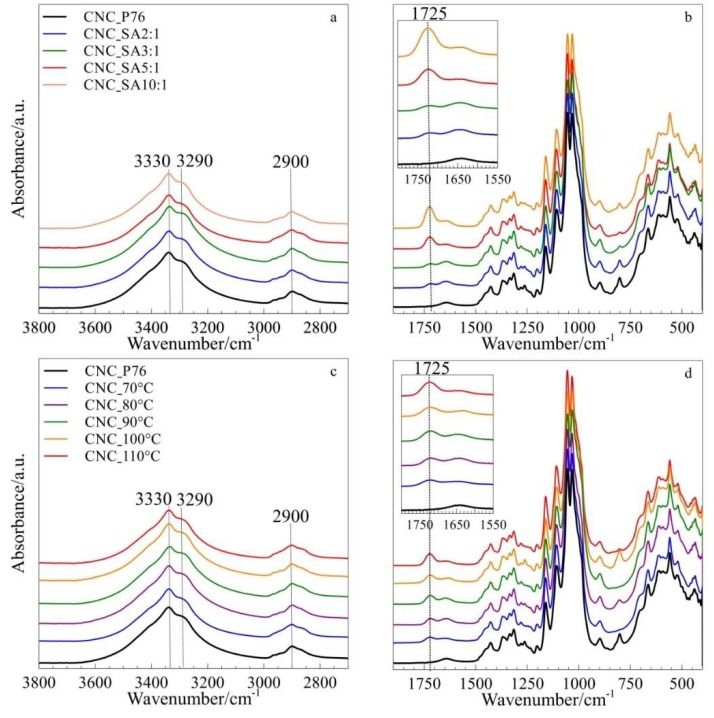

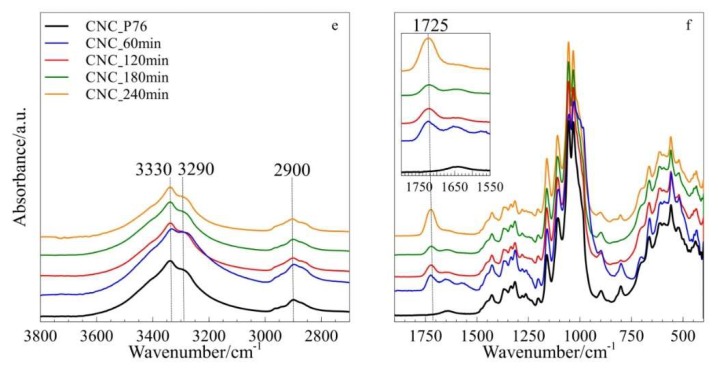
FTIR spectra of CNCs modified with succinic anhydride at various (**a**,**b**) SA:OH molar ratios; (**c**,**d**) temperatures and (**e**,**f**) times of reaction.

Figure 3 represents FTIR spectra of samples modified in various reaction conditions in comparison to the spectra of cellulose, hydrolysed in phosphoric acid solution. All spectra showed a characteristic broad band in the range of 3650–3000 cm^−1^, which is assigned to different O–H stretching modes. In cellulose I, a secondary hydroxyl group at the C3 and C2 position form a H-bond with an O5 and O6 atom of the contiguous ring, respectively. The first one appears at around 3342 cm^−1^, while the second one at 3432 cm^−1^. The band at 3277 cm^−1^ is characteristic for cellulose Iβ and is proportional to its amount [52,53]. The absorbance of methylene stretching bands, visible in the range of 2945–2857 cm^−1^, was shown to slightly increase after modification of cellulose with succinic acid anhydride, and thus can suggest a higher methylene group content after the esterification reaction [54]. All spectra displayed typical peaks at 1057 and 1162 cm^−1^ corresponding to C–O and C–O–C stretching vibration in cellulose, respectively [55,56], and a broad peak at 1643 cm^−1^ can be assigned to the bending vibrations of adsorbed water.

The partial esterification of cellulose hydroxyl groups was evidenced on FTIR spectra, for all modified samples, by the presence of the new band at 1725 cm^−1^. This absorption band is related to C=O stretching vibrations in ester bonds [57,58]. Its intensity increased with the increasing value of all three parameters, namely: SA:OH molar ratio, reaction time and reaction temperature, indicating a higher extent of CNC esterification. However, the molar ratio of reagents and reaction time had a prevailing effect on the esterification whilst the reaction temperature showed a moderate influence on the intensity of C=O stretching absorption band. The absence of bands at 1860 and 1790 cm^−1^, characteristic for succinic anhydride, confirms that the product is free from unreacted modifier [59].

### 3.3. XPS Analysis of Surface Chemical Composition of CNCs

**Figure 4:**
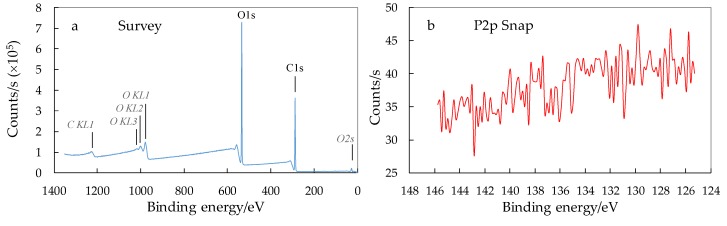
XPS spectra of CNC_P76: (**a**) XPS wide scan patterns; (**b**) XPS spectra in the range of binding energies of P 2p peak.

XPS spectra of CNC_P76 after hydrolysis revealed peaks for carbon and oxygen but no phosphorous was detected (Figure 4). For this sample, hydrolysis was carried out under conditions (phosphoric acid concentration and temperature) that were sufficient for the hydrolysis of amorphous regions without the activation of cellulose phosphorylation. The lack of phosphate groups bearing surface charges, on the as received nanocrystals, may also explain a slight tendency to form aggregates upon freeze-drying. On the other hand, the absence of phosphate groups was advantageous in terms of preserved thermal stability of CNC_P76, as discussed later.

**Figure 5:**
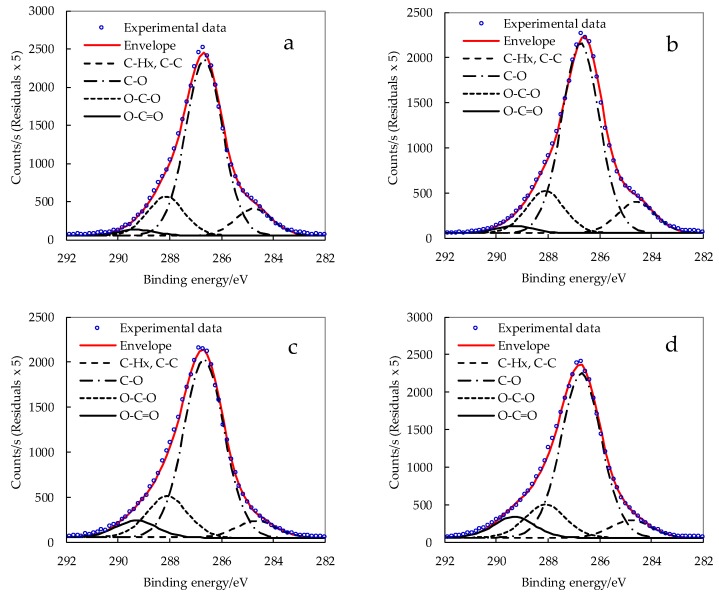
Resolved carbon C 1s peak for (**a**) CNC_P76 and surface modified CNCs at increasing molar ratio of succinic anhydride to cellulose hydroxyl groups: (**b**) CNC_SA3:1; (**c**) CNC_SA5:1; and (**d**) CNC_SA10:1.

The high degree of crystallinity, as determined from WAXD analysis and discussed later, indicated that nanoparticles retained intact crystalline core. Therefore, changes in chemical composition, due to succinylation of CNCs, are expected to occur at their surface. XPS, as a surface sensitive technique, was applied for evaluation of the degree of esterification. For cellulosic materials it collects information from an external layer with a maximum width of 10 nm [60], while approximately 2/3 of the signal originates from the first 3 nm on the surface [61]. The applied XPS method, as a surface sensitive technique, allowed for the changes in chemical constitution of an external layer of nanocrystals to be determined. However, it has limitations in terms of the evaluation of molecular changes in the core of nanoparticles, e.g., exposure of further –OH groups as a result of disruption/delamination of the crystalline structure. Carbon atoms, located in different molecular environments, display varied C 1s binding energies, which can be resolved from the high resolution XPS spectra to give qualitative and quantitative data. The binding energy of the carbon atom is shifted to higher values as the number of bonds with oxygen atoms increases. The high-resolution carbon C 1s signal showed four constituent peaks for all CNC samples (Figure 5). The peak at ∼284.8 eV is connected to carbon atoms without oxygen bonds (in C–C or C–H environment). The peaks with growing binding energy refer to carbon atoms with one (C–O, at ∼286.5 eV), two (O–C–O/C=O, at ∼288.6 eV), and three oxygen bonds (O–C=O, at ∼289.3 eV), respectively [62,63]. The C/O ratio was not influenced by the succinylation since the modifier introduces the same number of carbon and oxygen atoms (Table 2).

The indicative data concerning the degree of esterification was gathered from the resolved C 1s peak that displayed an increased percentage contribution of the O–C=O carbons in the modified CNC due to the formation of an ester bond. The determined surface substitution degree (SSD) tended to increase with growing molar excess of succinic anhydride. Interestingly, the trends in obtained substitution degrees were comparable to those reported by Liu et al. and Shang et al. for the homogeneous succinylation of cellulose in ionic liquids without catalyst [64,65]. Generally, homogeneous esterification of cellulose by succinic anhydride with catalyst resulted in significantly higher substitution degree [66]. In the case of succinylated nanocellulose, SSD values reported for esterified cellulose nanofibers took values comparable to our results [9,67]. It is believed that cellulose macromolecules with a high degree of substitution have reduced the possibility of hydrogen bonding in ordered crystal structures and tend to dissolve in solvent. Yuan et al. modified cellulose whiskers with less reactive alkenyl succinic anhydride (ASA) and the degree of substitution (DS) value, evaluated on the basis of XPS data, was 0.097 as opposed to 0.0158, determined by elemental analysis [68]. They demonstrated a more adequate determination of surface DS by XPS due to the local concentration of ester groups on the surface of nanocrystals.

### 3.4. Crystalline Structure of CNC

**Figure 6:**
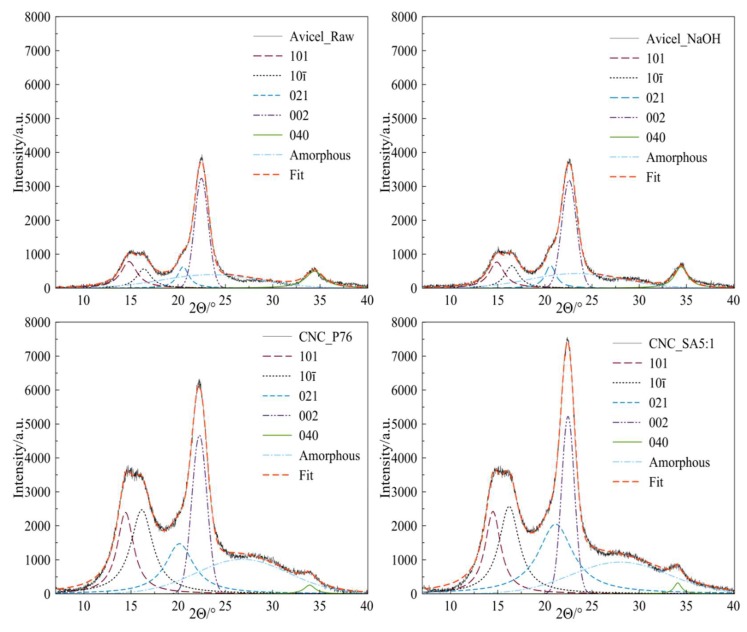
Representative deconvoluted WAXD patterns of raw Avicel, cellulose after alkali treatment, CNC obtained during hydrolysis in phosphoric acid solution (CNC_P76), and modified CNC (CNC_SA5:1).

Figure 6 presents the WAXD diffraction patterns of the raw material and representative modified samples. All the samples showed five crystalline peaks at 14.3°, 16.4°, 20.0°, 22.5° and 34.5° which corresponds to the 101, 10ī, 021, 002 and 040 Miller indices, characteristic for crystallographic planes of the monoclinic cellulose I lattice [48,49,50]. The broad reflection with the maximum at about 27.0° corresponds to the amorphous phase in the samples.

**Figure 7:**
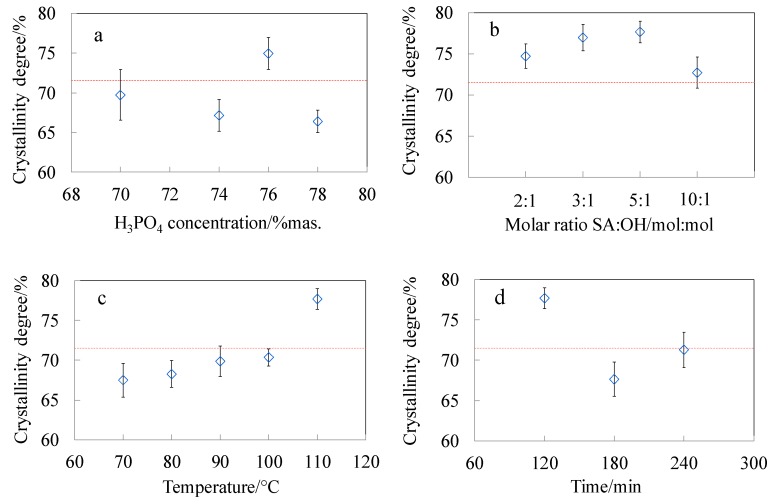
Crystallinity degree determined by deconvolution of WAXD patterns of (**a**) CNC_P series hydrolysed in phosphoric acid solutions at different concentrations; as well as CNC succinylated at different (**b**) SA:OH molar ratios; (**c**) temperature and (**d**) time. Dashed line depicts the crystallinity index of Avicel_Raw material.

One of the parameters which influences the yield of the hydrolysis, apart from the time and temperature, is the acid concentration [17,69]. Changes in the crystallinity degree of the CNC-P samples (Figure 7a) were small, for low concentrations of phosphoric acid solutions. The hydrolysis of amorphous domains was promoted when optimal acid concentration was achieved. The highest content of the crystalline fraction was observed for the sample hydrolysed in phosphoric acid with a concentration of 76 wt.%. Further increasing of the acid concentration resulted in a decrease of the crystallinity index. Hydrolysis of cellulose in acid solutions with a concentration above the optimal value, leads to the decrease of crystalline phase content as a result of hydrolysis of 1,4-β-glycosidic bonds in crystalline domains [4]. Moreover, level-off degree of polymerisation, which is obtained after the reduction of cellulose polymerisation degree by acid hydrolysis, correlates with the periodic crystal sizes. Severe reaction conditions may cause a significant decrease in the degree of polymerisation, and thus a decrease of crystal sizes [70,71].

The dependence of the crystallinity degree on the amount of succinic acid anhydride (Figure 7b) and reaction temperature (Figure 7c) was investigated in the course of CNC esterification. With the increasing values of both parameters the content of amorphous phase in the samples decreased. Such results indicate that mild hydrolysis proceeds during the modification reaction and these observations are in accordance with DLS analysis. After exceeding the optimal amount of the modifier (SA:OH 5:1), the crystalline phase content slightly decreased. However, there is no clear dependence of the crystallinity degree and the time of the reaction (Figure 7d). This effect can result from a high rate of hydrolysis in the initial stage of the reaction, when more easily available disordered domains are being attacked by the molecules of the modifier. The rate of hydrolysis decreases when the hydrolysis catalyst attacks the remaining, more resistant, crystalline domains [17]. The highest content of the crystalline phase (around 78%) was observed for the CNC_SA5:1 sample. In the course of CNC succinylation two chemical processes can be identified that influence the CNC crystal structure: (i) esterification of hydroxyl groups along with (ii) hydrolysis of 1,4-β-glycosidic bonds. As the molar ratio of SA:OH achieved the value of 10:1 the highest concentration of acidic catalyst could activate more extended hydrolysis of crystalline domains, thus lowering the cellulose crystallinity. Furthermore, the decreased crystallinity at the highest molar ratio can be correlated with the increasing esterification degree, as determined by the XPS analysis. This resulted in higher concentration of side chains that interfered the regular packing of cellulose macromolecules in crystals. The crystalline structure of CNC was not compromised by the extensive esterification and hydrolysis up to SA:OH molar ratio 5:1.

### 3.5. Microscopic Analysis of Cellulose Nanocrystals Morphology

**Figure 8:**
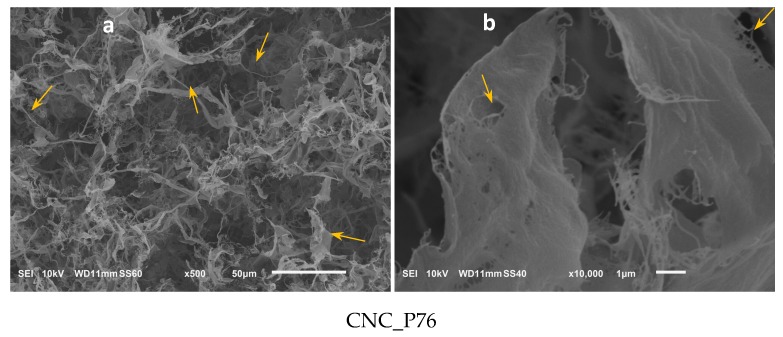

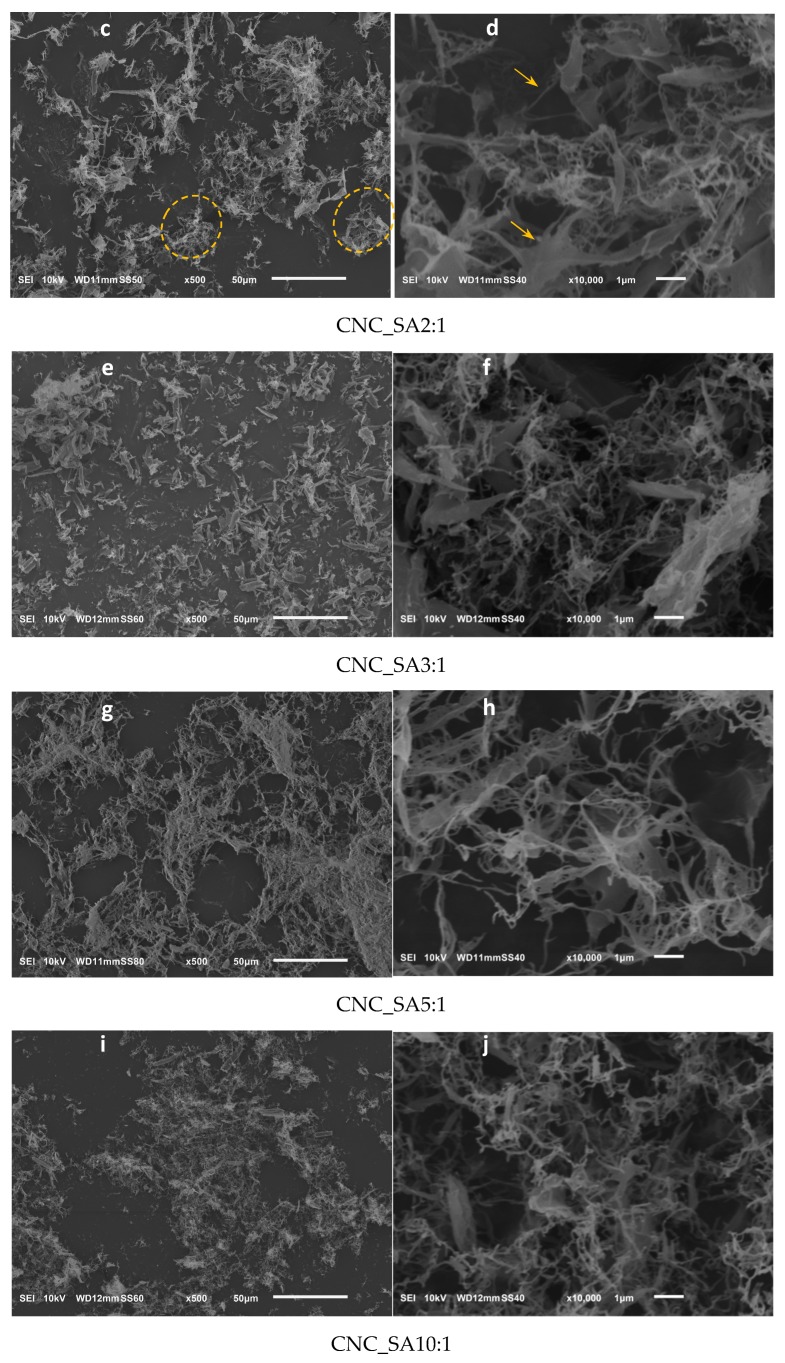
SEM images of (**a**,**b**) hydrolysed; and modified cellulose nanocrystals with different molar ratios of SA:OH: (**c**,**d**) CNC_SA2:1; (**e**,**f**) CNC_SA3:1; (**g**,**h**) CNC_SA5:1 and (**i**,**j**) CNC_SA10:1.

SEM photomicrographs of cellulose material obtained by hydrolysis in 76 wt.% phosphoric acid solution displayed microporous aerogel morphology. The material was composed of fibrous and ribbon-like aggregates marked on the photomicrograph with arrows (Figure 8a). The ribbons and membranes were formed by agglomerated fibrous structures with nanometric diameters. Since no signs of esterification by phosphoric acid were evidenced for this sample by FTIR and XPS analysis, the produced CNC were not protected from aggregation by repulsive interactions. 

Additionally, Zhang et al. have investigated the influence of the acid type on the morphology of cellulose nanocrystals, and showed that during the hydrolysis in phosphoric acid, less hydrogen ions attack amorphous regions, and thus hydrolysis in H_3_PO_4_ results in the production of larger particles than those from commonly used sulphuric acid [14].

Better individualisation of the nanoparticles was observed after their modification with succinic anhydride (Figure 8c–j). The presence of succinate groups on the surface of nanocrystals played a role in structure formation of aerogel, probably by steric hindrance and repulsive interactions of negatively charged carboxylic end groups that limited agglomeration. 

Reduction of the agglomeration process was additionally supported by the use of tert-butanol while freeze-drying, which can provide steric hindrance and prevent hydrogen bond formation between nanoparticles. It results from the structure of the tert-butanol molecule, which consists of three methyl and one hydroxyl groups, and can form hydrogen bond with only one water molecule or one hydroxyl group on the surface of CNC [46,72].

Morphological studies of freeze-dried modified cellulose nanocrystals showed that in the micrometric scale they take a form of loose aerogel or powder with highly developed surface area (Figure 8c,e,g,i). The irregularly shaped microparticles were composed of high number of sub-micrometre membranes, ribbons and nanofibers (Figure 8d,f,h,j). The flat elongated agglomerates, a few micrometres in length and diameter on the nanoscale, become less dominant while the nanofibrillar aggregates became prevailing as the molar ratio of the succinic anhydride to cellulose hydroxyl groups increased. The sample CNC_SA10:1 showed basically nanofibrous morphology (Figure 8j). Such results are in accordance with the CNC characteristic dimensions given by Moon et al. [73]. Moreover, the presence of particles with nanometric and submicronic dimensions was observed for all the samples, but with the increasing molar ratio of SA:OH, the contribution of submicronic particles decreased.

**Figure 9:**
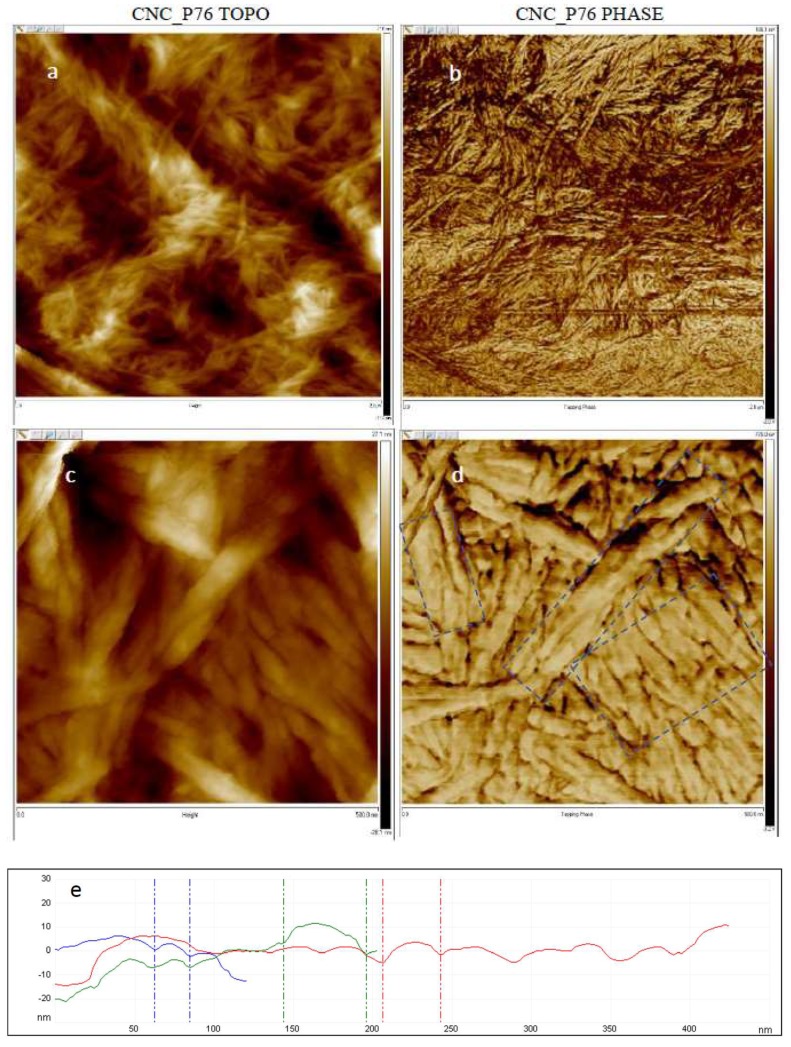

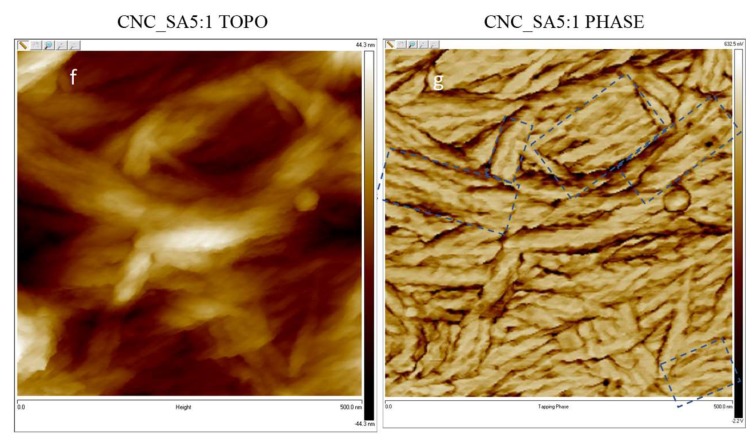
Representative AFM topography and phase images of CNC-P76 at scanning areas of (**a**,**b**) 2 × 2 μm, and (**c**,**d**) 500 × 500 nm, and (**f**,**g**) CNC-SA 5:1 at scanning area of 500 × 500 nm samples; (**e**) cross-sectional profiles taken from the CNC-P76 topography image.

AFM scans of CNCs dried on air showed similar morphology before and after surface modification (Figure 9). Both esterified and pristine CNCs had tendency to form randomly oriented agglomerates of aligned nanoparticles. The typical areas of parallel cellulose nanocrystals forming aggregates were marked with rectangles on the phase images (Figure 9d,g). The nanocrystals showed a tendency to align along the long edges and pack tightly when dried on air. The AFM observations revealed strong surface tension of CNCs leading to agglomeration regardless of the surface modification. That shows the necessity to apply special drying procedure, such as freeze-drying with addition of organic solvents, in order to preserve the nanoparticulate morphology of the produced CNCs. The average diameter of the nanocrystals in the referential CNC-P76 sample was 33.0 nm with a standard deviation of 9.4 nm, while for succinylated CNC (CNC_SA5:1) those parameters took values of 29.2 nm and 9.5 nm, respectively. Esterification had a moderate influence on the nanocrystals diameters. A slight decrease in the dimensions of succinylated CNCs, that was evidenced by AFM and DLS measurements, indicated mild hydrolysis of cellulose occurring at the surface of nanoparticles in the course of esterification.

### 3.6. Studies of Pyrolytic and Thermo-Oxidative Degradation of CNCs Obtained by Hydrolysis and Surface Esterification

**Figure 10:**
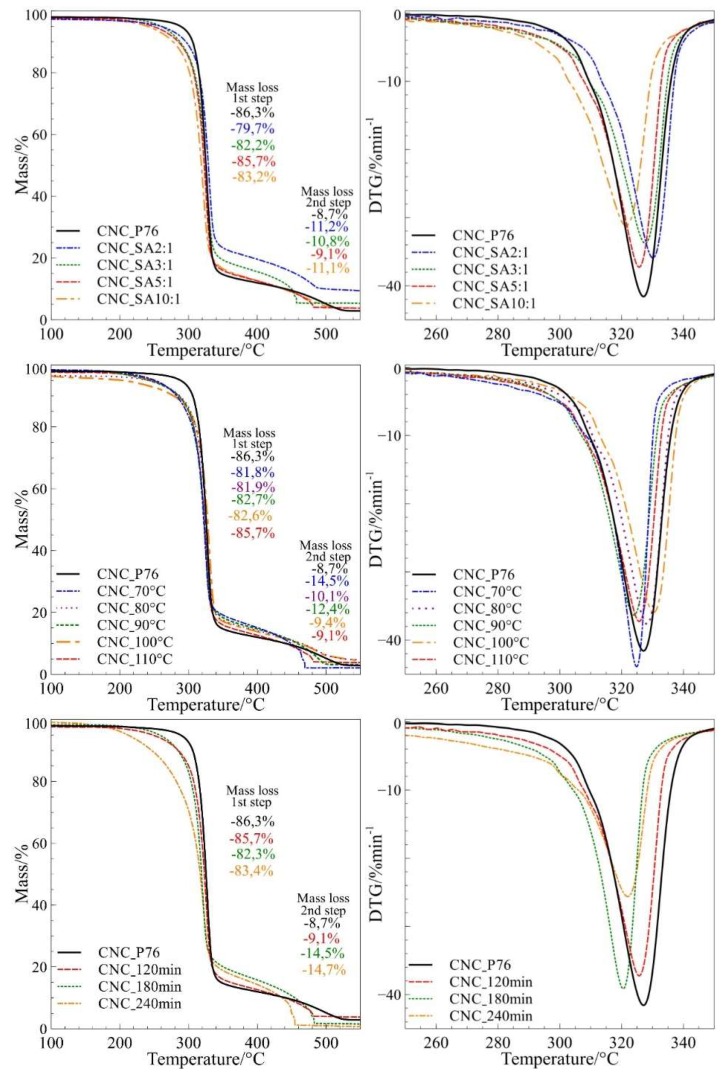
TG and DTG curves of modified cellulose nanocrystals during thermo-oxidative degradation.

Thermogravimetric analysis was performed in the temperature range of 30–600 °C for both argon and air atmospheres. Figure 10 presents TG and DTG curves for modified samples recorded in synthetic air atmospheres. The TGA curves on the graphs were presented in narrower temperature range for better presentation of differences in the course of mass loss during the main steps of degradation. The characteristic degradation parameters of all cellulosic materials are shown in Table 3. The thermal stability of cellulose hydrolysed in phosphoric acid with different concentrations showed, that the acid concentration does not have a significant influence on the thermal stability of CNCs prepared from Avicel, until the optimum concentration (76 wt.%) is exceeded.

**Figure 11:**
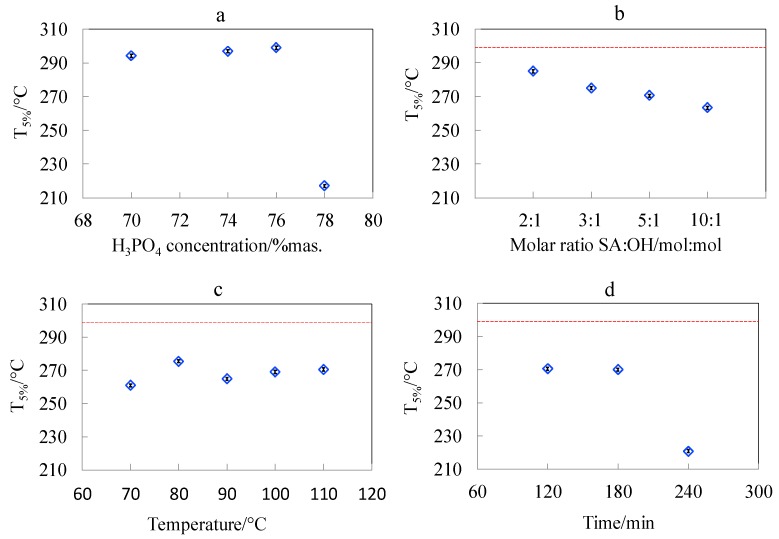
Dependence of the initial temperature of thermo-oxidative degradation (T_5%_) of cellulose nanocrystals (**a**) obtained during hydrolysis at different concentrations of phosphoric acid; and succinylated cellulose nanocrystals obtained at increasing (**b**) SA:OH molar ratio, (**c**) temperature, and (**d**) time of reaction. Dashed line depicts the T_5%_ value for the CNC_P76 nanocrystals subjected to surface esterification.

It was demonstrated that CNC bore no phosphate groups when produced in phosphoric acid at concentration of 76 wt.% (Figure 4a,b) which was crucial for preserving the good thermal stability of CNC. Increasing of the phosphoric acid concentration from 76 wt.% to 78 wt.% caused a tremendous decrease of the initial degradation temperature (T_5%)_ from 285.6 °C to 233.6 °C (by 52 °C) (Figure 11a). This observation allows the determination of hydrolysis parameters that preserve the thermal stability of cellulose nanocrystals on the initial level, and is crucial for the foreseen application of CNC as a filler for engineering biopolymers with processing temperature over 230 °C. 

The application of Avicel microcrystalline cellulose as a raw material allowed to produce thermally stable cellulose nanocrystals by the phosphoric acid catalysed hydrolysis. The outcome from our earlier work [4], where a different raw material was used, and current results demonstrate the effect of raw material on the thermal stability of hydrolysis product. When UFC 100 was used as a raw material the reduction of thermal stability was already observed at the phosphoric acid concentration of 74 wt.% despite the product was hydrolysed at lower temperature (90 °C) and preserved its microcrystalline morphology. The initial temperature of degradation (T5%) for this material was 219.5 °C. On the other hand, for the Avicel cellulose used in this work the initial temperature of degradation took the significantly higher value of 285.6 °C for the sample hydrolysed in 76 wt.% phosphoric acid solution and at 100 °C. Different commercial microcrystalline celluloses used as a feedstock showed variation in thermal stability of hydrolysis products probably due to different natural origin and processing history.

All modified samples were characterised by slightly lower thermal stability as compared to cellulose nanocrystals from 76 wt.% phosphoric acid. The investigations on the influence of SA:OH molar ratio on thermo-oxidative and pyrolytic degradation of cellulose nanocrystals showed that with the increasing amounts of succinic anhydride, the thermal stability of samples decreased in both air and argon atmospheres. The systematic decrease of T_5%_ with the increasing SA:OH molar ratio in the course of thermo-oxidative degradation is presented in Figure 11b. The onset temperature of pyrolytic degradation (T_onset_) for the CNC_SA2:1 sample was 316.3 °C, while for CNC_SA10:1 it decreased to 303.6 °C. According to Sehaqui et al., the lower thermal stability of carboxylated CNC samples may result from their higher surface area, which results in a lower resistance to thermal degradation [35]. 

There was no clear dependence of thermal resistance of modified cellulose nanocrystals on the reaction temperature (Figure 11c), however, the highest thermal stability, with regards to T_onset_, was observed for the sample modified at 100 °C. The onset temperature for this sample was 340.1 °C and 315.5 °C in argon and air atmospheres, respectively. Investigations of the influence of reaction time on the thermal behaviour of succinylated CNC showed that there are no significant differences between the thermal stability of samples modified for 120 and 180 min. However, when the reaction was carried out for 240 min, the resulting sample had apparently lower values of T_5%_ as compared to samples modified at shorter reaction times (Figure 11d). The variation of initial temperature of degradation (T_5%_) indicated that succinylation generally caused a decrease of CNC thermal stability, especially in an oxidative atmosphere. However, the highest reduction of thermal stability, with substantial mass loss starting at around 200 °C, was observed for the longest reaction time (240 min). The stability of modified nanocrystals was higher in an inert atmosphere. This observation has a practical meaning for the application of modified CNC as a filler in polymer nanocomposites. The degradation can be prevented by feeding the nanofiller to an already melted polymer. This could reduce the exposure of CNC-SA to the detrimental oxygen action by immediate blending. Therefore, by a proper selection of succinylation parameters and processing procedure, one can achieve a narrow but stable processing window for biopolymer/succinylated CNC nanocomposites. The differences in the thermal stability of cellulosic samples may result not only from their surface area, but also from their degree of polymerisation. Samples with lower degrees of polymerisation have lower thermal stability, and therefore, with the increasing time of modification reaction, the thermal stability of CNCs decreased [35]. 

Furthermore, apart from the surface area and a degree of polymerisation, the thermal stability of cellulose nanocrystals is also influenced by the degree of substitution. The correlation of thermogravimetric studies with the results obtained from XPS analysis indicated that the thermal stability of nanoparticles decreases with the increasing degree of substitution. According to investigations provided by Trinh et al. and Wang et. al, this effect may be associated with the partial substitution of –OH groups in cellulose by succinic anhydride and thus, incorporation of free carboxylic group on the CNCs surface. Acidic groups can catalyse the dehydration process, and therefore, shift the beginning of the degradation towards lower temperatures [74,75].

The overall characteristics of produced CNCs, that are considered as a potential filler for bioplastics, indicated a critical phosphoric acid concentration during hydrolysis (76 wt.%) as well as SA:OH molar ratio (5:1) in the course of esterification. Exceeding the critical parameters resulted in reduction of thermal stability required for composite processing. The decrease in thermal stability of produced CNCs could arise from both chemical and structural changes of cellulose, such as incorporation of significant amount of less thermally stable ester groups of inorganic or organic acid, extensive hydrolysis as well as reduction in crystallinity. On the other hand CNCs succinylated at the highest SA:OH molar ratio 10:1, offered the finest nanoporous morphology, as revealed by SEM, combined with the highest substitution degree, evidenced by XPS and FTIR analysis, and well preserved crystallinity. This material would fulfil requirements of functional applications, among others, filtration membranes, sorbents, sensors, drug carriers, etc. The highest applied esterification temperature (110 °C) could be recommended for the synthesis of succinylated CNCs. The increase of temperature had no significant effect on the thermal stability of whiskers while it promoted growth of CNCs’ crystallinity. High crystallinity, and thus intrinsic stiffness, along with high aspect ratio are desired features of the reinforcing nanofiller. Generally, the selection of manufacturing parameters would depend strictly on the foreseen application of CNCs and demanded essential properties.

## 4. Conclusions

Cellulose nanocrystals were produced by hydrolysis in phosphoric acid solutions at different concentrations. The optimal acid concentration that allowed effective hydrolysis of amorphous regions, without activation of cellulose phosphorylation, was found at 76 wt.%. The nanocrystals produced in optimal conditions had a high crystallinity index of 74% and preserved thermal stability. The influence of reaction time, temperature, and molar ratio of the reagents in the course of heterogeneous esterification of cellulose nanocrystals by succinic anhydride was further investigated. The surface esterification of CNC was confirmed by FTIR and XPS. The partial hydrolysis of cellulose, along with the esterification reaction, was indicated by the reduction of average particle size from the length of approximately 100 nm for CNC_P76 to 60 nm for the succinylated CNC. The surface substitution degree of esterified CNCs clearly increased with the increasing molar ratio of SA:OH and varied from 0.10 to 0.32. Significant reduction of thermal stability of modified CNCs was observed only for the highest substitution degree and the longest time of reaction (240 min). By the controlling of succinylation parameters, one can manufacture CNCs with desired sizes, extent of esterification or preserved thermal stability. The elaborated method of CNC functionalization by using succinic anhydride makes it possible to fabricate compatible and thermally stable cellulose nanocrystals that can be applied as a reinforcement in polymer composites. Such a study, utilizing selected polyamides, is now in progress.

## Figures and Tables

**Table 1:** Description of prepared samples.

**Sample Denotation**	**Hydrolysis Catalyst**	**Concentration of Acid Solution,** **wt. %**	**Time,** **min**	**Temperature,** **°C**
Avicel _Raw	-	-	-	-
CNC_P70	H_3_PO_4_	70.0	90	100
CNC_P74		74.0		
CNC_P76		76.0		
CNC_P78		78.0		
**Sample Denotation**	**Modifier**	**Molar Ratio of SA:OH**	**Time,** **min**	**Temperature,** **°C**
CNC_SA2:1	Succinic anhydride	2:1	120	110
CNC_SA3:1		3:1		
CNC_SA5:1		5:1		
CNC_SA10:1		10:1		
CNC_ 70 °C	Succinic anhydride	5:1	120	70
CNC_80 °C				80
CNC_90 °C				90
CNC_100 °C				100
CNC_110 °C				110
CNC_60 min	Succinic anhydride	5:1	60	110
CNC_120 min			120	
CNC_180 min			180	
CNC_240 min			240	

**Table 2:** Atomic concentrations from XPS spectra and a resolved carbon C 1s peak for alkali treated cellulose (Avicel NaOH), CNCs obtained in phosphoric acid solution with concentration of 76 wt.% (CNC_P76) and surface modified CNCs at different molar ratio of succinic anhydride to cellulose hydroxyl groups.

Sample	O [%]	C [%]	C/O [%]	Resolved C 1s Peak				Surface Substitution Degree (SSD)
				C–H_X_ [%]	C–O [%]	C=O/O–C–O [%]	O–C=O [%]	
Avicel_NaOH	43.0	57.0	1.3	3.9	41.2	9.9	1.9	0.12
CNC_P76	42.0	58.0	1.4	6.3	41.1	9.1	1.4	0.08
CNC_SA2:1	41.5	56.6	1.4	6.4	40.9	8.4	1.9	0.12
CNC_SA3:1	41.9	56.6	1.4	6.7	40.2	8.9	1.6	0.10
CNC_SA5:1	43.0	56.5	1.3	3.8	39.9	9.2	3.7	0.23
CNC_SA10:1	42.6	56.7	1.3	4.4	39.7	8.0	5.1	0.32

**Table 3:** Parameters of pyrolytic and thermo-oxidative degradation of cellulose materials.

Sample	Pyrolytic Degradation				Thermooxidative Degradation			
	T_5%_, °C ± 0.4 °C	T_onset_, °C ± 2 °C	T_max_, °C ± 2 °C	Char % * at 600 °C	T_5%_, °C ± 0.4 °C	T_onset_, °C ± 2 °C	T_max_, °C ± 2 °C	Char % * at 600 °C
Avicel-Raw	300.7	314	335	8.13	293.0	303	320	2.10
Avicel-NaOH	281.6	335	363	10.72	290.0	320	333	1.84
CNC_P70	285.6	338	365	10.44	294.2	315	327	2.15
CNC_P74	288.9	336	357	7.30	297.0	315	327	3.46
CNC_P76	285.6	332	354	4.60	299.0	314	327	2.84
CNC_P78	233.6	300	338	14.18	217.0	247	308	14.57
CNC_SA2:1	286.4	340	367	7.23	285.1	316	330	8.70
CNC_SA3:1	262.4	334	367	6.87	275.1	311	328	4.90
CNC_SA5:1	260.1	342	373	6.57	270.6	311	326	2.80
CNC_SA10:1	258.1	327	358	5.56	263.4	304	322	2.98
CNC_ 70 °C	260.4	331	364	7.50	261.0	312	325	2.05
CNC_ 80 °C	277.1	340	364	5.04	275.6	314	328	4.46
CNC_ 90 °C	269.1	333	361	7.26	264.9	309	324	3.04
CNC_ 100 °C	282.9	340	371	6.86	269.1	316	330	3.78
CNC_ 110 °C	260.1	342	373	6.57	270.6	311	326	2.80
CNC_ 120 min	260.1	342	373	6.57	270.6	311	326	2.80
CNC_ 180 min	260.9	334	365	6.37	270.1	306	321	1.44
CNC_ 240 min	212.1	330	356	7.68	220.9	300	322	0.66

* The relative error in the measurement of char residue is c.a. 0.003.
